# Repeat Point-of-Care Echocardiographic Evaluation of Traumatic Cardiac Arrest: A New Paradigm for the Emergency Physician

**DOI:** 10.5811/cpcem.2017.2.33021

**Published:** 2017-05-23

**Authors:** Benjamin Thomas, Edward Durant, Sophie Barbant, Arun Nagdev

**Affiliations:** * Highland General Hospital, Department of Emergency Medicine, Oakland, California; † Highland General Hospital, Department of Cardiology, Oakland, California

## Abstract

We report a case of a 52-year-old man who presented to the emergency department (ED) in extremis (hypotensive with an altered sensorium) with subsequent cardiac arrest after a motor vehicle collision. The initial trauma evaluation did not reveal a source of the hemodynamic compromise. A point-of-care ultrasound revealed severe mitral regurgitation secondary to an anterolateral papillary muscle rupture. Patient underwent successful emergent mitral valve replacement after initial resuscitative efforts and intraaortic balloon pump placement.

## INTRODUCTION

**Image 1:**
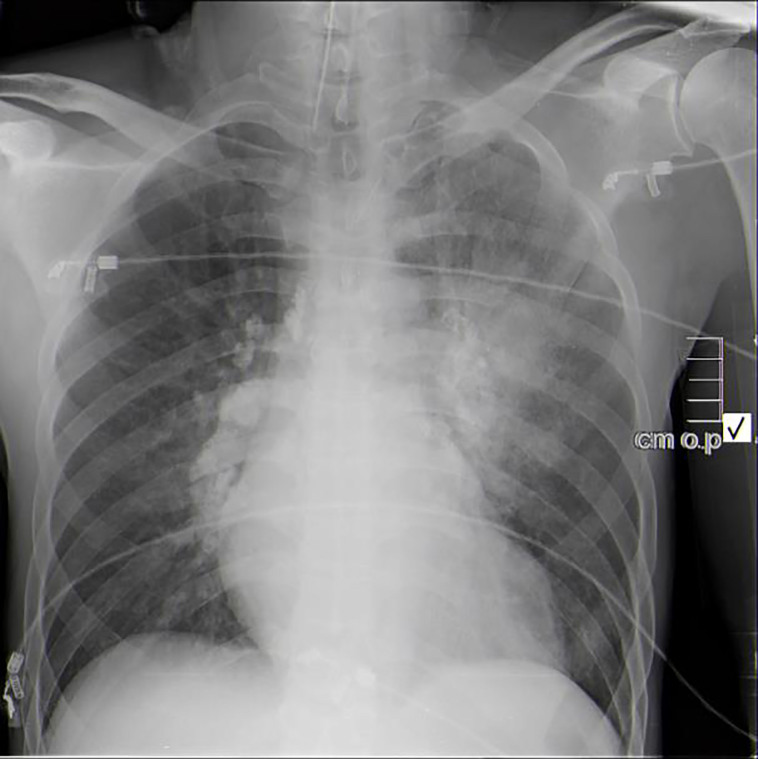
Anterior posterior chest radiograph demonstrates unilateral (left sided) opacities.

A 52-year-old man with an unknown history was transported in extremis by emergency medical services after a severe motor vehicle collision (MVC) into the side of a home. On arrival to the emergency department (ED), the patient’s vital signs were blood pressure 90/50 mmHg, heart rate 110 beats per minute, respiratory rate 12 breaths per minute, oxygen saturation 95% on high flow non-rebreather mask, and a Glasgow coma score (GCS) of 8. During the primary survey, the patient was intubated due to labored breathing and a low GCS. The initial primary and secondary surveys did not reveal any signs of gross injury: his pelvis was stable, no long bone injuries were present, and rectal tone was intact prior to intubation. The initial extended focused assessment with sonography in trauma (EFAST) examination did not reveal free fluid in the chest, abdomen, pericardium, or pelvis. A modified pulmonary point-of-care ultrasound (POCUS) examination did not demonstrate a pneumothorax in either side of the chest, but did reveal significant B-lines in the left upper lung field that correlated with an AP chest radiograph (Image 1).

The pelvis was stabilized with a pelvic binder because of the persistent hypotension, and four units of packed red blood cells were transfused via a Level 1 infuser. An electrocardiogram (ECG) revealed sinus tachycardia with frequent ectopy, left axis deviation, right bundle branch block, ST depressions and T-wave inversions in multiple leads (anterior/septal, lateral) and hyperacute T waves in the inferior leads without obvious ST elevation, all of which were new compared with a prior ECG for the patient.

The patient was rapidly taken to computed tomography (CT) imaging because a clear source of hypotension could not be identified. After imaging, the patient was found to be in ventricular fibrillation, resulting in cardiac arrest as identified on a portable cardiac monitor, with a corresponding loss of pulses. Cardiopulmonary resuscitation and defibrillation were initiated and the patient regained spontaneous circulation within two minutes. CT revealed multifocal airspace consolidations, predominantly in the left upper lobe and right lower lobe, with no evidence of head, chest, or abdominal injury. Upon return to the trauma bay, a repeat POC echocardiogram was performed demonstrating hyperdynamic LV systolic function, severe mitral regurgitation (MR), and a ruptured anterolateral papillary muscle (Image 2A, 2B). An urgent cardiology consultation confirmed the POC echocardiography findings, and a diagnostic catheterization revealed no evidence of coronary occlusion. An intraaortic balloon pump was placed and the patient was emergently transferred to a surgical center for mitral valve replacement. The mitral valve replacement with a bioprosthesis was uncomplicated. Two weeks after the initial incident the patient was discharged with no cardiac or neurologic deficits, and followed up with cardiology services.

CPC-EM Capsule

What do we already know about this clinical entity?

The goal of POCUS in blunt trauma patients is to detect significant free fluid in the abdomen/thorax, and/or pericardial space and/or a significant pneumothorax.

What makes this presentation of disease reportable?

POCUS has utility in detecting more subtle findings (specifically cardiac) that are unrecognized during the initial resuscitation of the critically ill trauma patient.

What is the major learning point?

Once the classic findings of trauma are ruled out in the unstable trauma patient, repeat the POCUS evaluation looking for gross valvular abnormalities.

How might this improve emergency medicine practice?

As clinicians become more facile with POCUS, important findings can be detected during the initial resuscitation of the critically ill trauma patient.

## DISCUSSION

Myocardial contusion is the most frequent cardiac injury observed after blunt chest trauma.^1^ Valvular injury is less common. The aortic valve is the most frequently injured, followed by the mitral and tricuspid.^2^ Acute MR due to papillary muscle rupture is a known complication of acute myocardial infarction. However, there are only a few cases of acute rupture secondary to blunt chest trauma, and no reported cases detected by POC echocardiography during the initial trauma resuscitation. Due to higher rates of MVCs, traumatic valvular rupture rates have increased, with clinical presentation varying from asymptomatic to cardiogenic shock.^3^,^4^

The mechanism of injury is thought to be due to the compression of the heart from sudden deceleration and transfer of kinetic energy to the patient’s chest during late diastole. During this time in the cardiac cycle, the chambers are full and the valves are closing or closed. The valves are most vulnerable at this point to deceleration or compression injury.^5^–^7^ On physical exam, the intensity of the murmur does not necessarily correlate with the severity of the murmur. Some patients with severe MR attributable to rupture have early equalization of left ventricular and left atrial pressures, resulting in silent MR (as in our patient) or a relatively soft, short, and indistinct murmur in as many as 50% of patients.^8^

One of the key diagnostic findings in this case was the presence of unilateral pulmonary edema (UPE) on the initial chest radiograph and POCUS. In a recent large, retrospective European study of patients referred to the intensive or coronary care units with cardiogenic shock, UPE was invariably associated with severe MR.^9^ Although the finding of right-sided UPE was much more common, prolapse of the anterior leaflet, as was the case with our patient, was associated with left-side UPE.

POCUS has altered the framework for evaluating and managing the unstable trauma patient. EFAST evaluation allows for a directed evaluation of the abdomen, chest, and pericardium, which are cavities that can accumulate blood and cause hemodynamic compromise.^10^ It can also be vital in the detection of a large pneumothorax that may prevent venous return to the heart. Conversely, a negative EFAST examination compels the clinician to look for other sources of hemodynamic decompensation (e.g. retroperitoneum, long bone fractures, spinal injury, etc.). A negative EFAST examination (with a negative CT of the chest and abdomen for hemorrhage) in a critically ill trauma patient should trigger the astute clinician to search for the underlying acute pathology that may have preceded the trauma. Given our experience, a detailed POC echocardiographic evaluation looking for ejection fraction, right ventricular size and gross valvular function has become our de facto protocol.

Papillary muscle rupture is a surgical emergency, and we conclude that the emergency physician’s repeat, detailed POC echocardiography exam after the negative chest and abdomen CT in this patient prevented complete cardiac collapse. The emergency physician should entertain other sources of shock (cardiogenic, hypovolemic, distributive) in these difficult cases Using POCUS is even more critical when initial standard imaging studies fail to find the source of the hemodynamic compromise. Without POCUS, our patient likely would not have survived the acute cardiac insult, reinforcing our belief that POCUS evaluation of the critically ill patient is imperative during both the initial trauma evaluation, and also during the re-evaluation when a clear source of shock is not detected.

## Supplementary Information



## Figures and Tables

**Image 2A:**
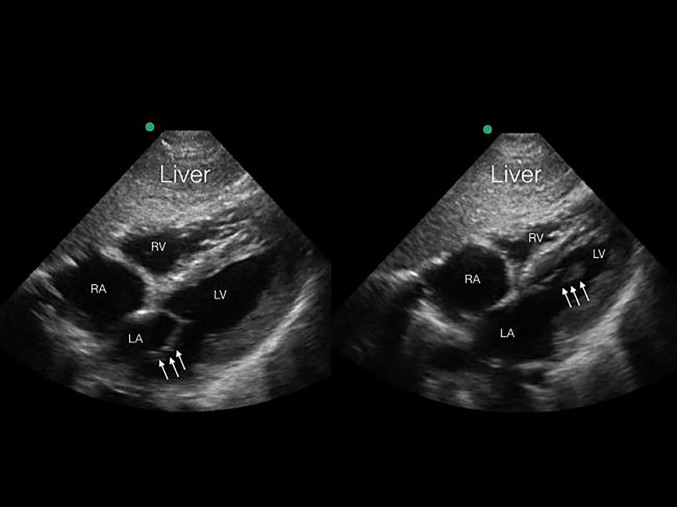
A subxiphoid four chamber view of the heart demonstrates the mobile detached papillary muscle (arrows). *RA*, right atrium; *RV*, right ventricle; *LA*, left atrium; *LV*, left ventricle.

**Image 2B:**
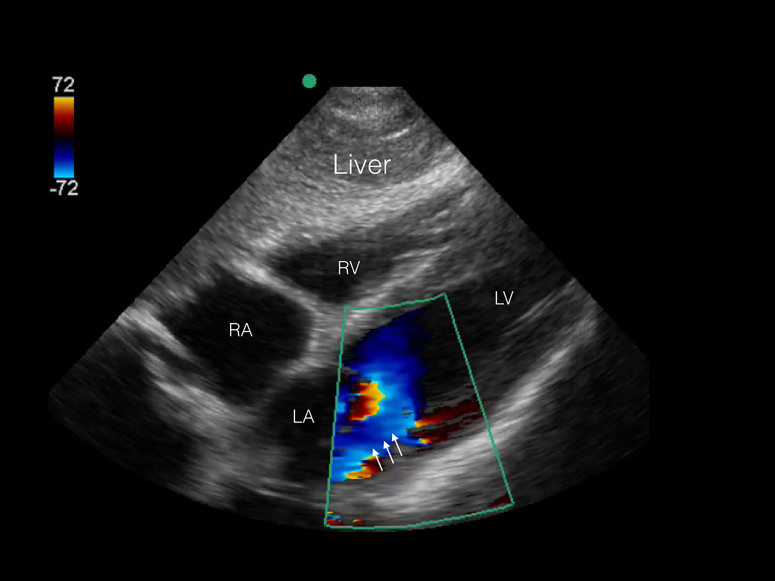
A subxiphoid four chamber view of the heart with color Doppler demonstrates a severe mitral regurgitation jet (arrows). *RA*, right atrium; *RV*, right ventricle; *LA*, left atrium; *LV*, left ventricle.
